# Adaptive Digital Twin Modeling with Control: Integration of Extended Kalman Filter-Based Recursive Sparse Nonlinear Identification with Model Predictive Control

**DOI:** 10.3390/s26051734

**Published:** 2026-03-09

**Authors:** Jingyi Wang, Liang Cao, Yankai Cao, Bhushan Gopaluni

**Affiliations:** 1 Department of Chemical and Biological Engineering, University of British Columbia, Vancouver, BC V6T 1Z3, Canada; wjy1129@student.ubc.ca; 2 Department of Chemical Engineering, Massachusetts Institute of Technology, Boston, MA 02139, USA; liangcao@mit.edu

**Keywords:** adaptive algorithms, digital twins, extended Kalman filter, sparse matrices, model predictive control

## Abstract

The adoption of digital twins has revolutionized industrial process simulation, monitoring, and control effectiveness. However, practical implementations of digital twins are hindered by substantial challenges, including extended development time, diminishing model accuracy, and restricted interactive capabilities. Addressing these critical issues, this paper proposes a comprehensive digital twin development framework that integrates digital twin identification, real-time model updating, and advanced process control. The proposed approach first identifies the offline digital twin model through the sparse identification of a nonlinear dynamics algorithm, reducing the digital twin development time while maintaining model fidelity. Then, the identified model is updated by the extended Kalman filter to mitigate the problem of diminishing accuracy. Finally, incorporating the latest updated model into the model predictive control facilitates the control inputs optimization and enhances the interactive capacity of digital twins. Through one industrial case study and two simulation examples, the advantages of the proposed algorithm are demonstrated.

## 1. Introduction

With ever-expanding technological innovation, the concept of digital twins has emerged as a revolutionary paradigm that connects physical and digital domains [1,2]. By continuously collecting and integrating data from various sources, digital twins not only provide an accurate real-time reflection of the physical entity but also facilitate the process control effectiveness. The growth of advanced industrial internet platforms that integrate artificial intelligence into industrial software is driving the widespread adoption of digital twins, which allows enhanced process simulation, state prediction, and input optimization across various industries [3,4,5].

From a foundational cyber-physical paradigm to a specialized control-theoretic tool, the concept of the digital twin represents a hierarchical instantiation of model-based systems engineering. At its most abstract, a digital twin constitutes a dynamic, data-driven virtual representation of a physical entity, synchronized across its lifecycle to enable simulation, analysis, and control. This paradigm bridges the virtual and physical through bidirectional data flows.

Within industrial systems, this manifests as the process digital twin: a first-principles or hybrid model of a continuous or batch production system. It emulates the underlying physics, chemistry, and unit operations to serve as a high-fidelity predictive and diagnostic platform for process design, optimization, and operator training. Its most operationally acute form is the process control digital twin. This is a real-time, closed-loop implementation tightly coupled with the plant’s distributed control system (DCS). Functioning as a soft sensor and a predictive model within the control hierarchy, it facilitates advanced process control (APC) and real-time optimization (RTO) [6].

Since its original development, the application area of digital twins has expanded from aerospace to information and communications technology (ICT), as well as manufacturing [7,8]. In [9], a digital twin framework for smart manufacturing was proposed using deep reinforcement learning to perform online optimization for reconfiguration planning. Additionally, in [10], the digital twin for a painting robot was developed to enable painting process practices without consuming physical assets. Furthermore, a modular framework was developed in [11] to implement a flexible and cost-effective digital twin for the power system.

A comprehensive industrial digital twin circle is a smooth combination of different aspects, which include the underlying algorithm that supports simulation, prediction, and control optimization, the front-end 3D visual modeling for user interactions, and the backend system for data acquisition and programmable logic controller (PLC) command dispatch. Among these aspects, robust and accurate models build the foundations for digital twins to realize their potential to ensure fidelity, synchronization, and effective interactions between the physical and virtual spaces [12]. As a result, this research focuses on the algorithm perspective of the digital twin to streamline the digital twin’s model identification procedure and integrate the digital twin model with advanced process control.

Striking a balance between model accuracy and complexity is of utmost importance in digital twin identification to prevent overfitting and reduce computational cost [13]. A sparse and structured, function-on-function model was proposed in [14] to hierarchically select the significant variables for digital twin model constructions. Moreover, an adaptive, sparse graph learning approach was proposed in [15], using a digital twin model to generate the performance degradation data, achieving accurate remaining useful life (RUL) prediction for rolling element bearings. Among the sparsity-promoting identification algorithms, the sparse identification of nonlinear dynamics (SINDy), introduced in [16], has gathered increased scholarly interest in recent times. It employs a three-step, sparse regression framework to automatically determine the governing equations for dynamic systems. The current absence of a standardized modeling framework for digital twins necessitates a substantial investment of research effort in methodological selection. The SINDy-based algorithms address this gap by offering a streamlined and principled development pipeline, thereby significantly reducing the time required for model construction. Furthermore, its core methodology inherently performs a trade-off between model complexity and predictive accuracy, automatically selecting a parsimonious set of governing dynamics. This ensures that the derived model achieves a high degree of congruence with the true underlying system behavior.

Real-time synchronization is another critical aspect in digital twin algorithm developments, essential for maintaining accuracy over extended periods. To achieve this, adaptive identification is required to perform online model updates while simultaneously minimizing computational burdens. The Kalman filter and its variants, renowned for their recursive estimation nature and capacity to handle system uncertainties, have been adapted to estimate parameters within the system model, facilitating the identification and calibration of models in real time. In [17], a Kalman filter and SINDy integrated algorithm was proposed to perform online-adaptive, multi-input, multi-output (MIMO) digital twin identifications, offering a dynamic and responsive solution for digital twin modeling in complex systems. However, this algorithm only addresses the modeling and synchronization aspects of digital twin development, disregarding the interactive functionality that allows users to control the real physical operational performance by manipulating the digital interface of digital twins [13].

To facilitate effective interactions between user directives and system operations, it is imperative to integrate advanced process control algorithms into the development of digital twins. The model-predictive control (MPC) plays a significant role in the field of advanced process control and has been applied extensively in various industries, including automobile controlling [18] and urban system controlling [19]. By incorporating external inputs into SINDy’s framework, the SINDy with control (SINDYc) algorithm was introduced in [20]. This algorithm was then integrated with the MPC to establish a dependable framework for concurrently realizing the modeling and interaction functionalities of digital twin algorithms. However, based on a time-invariant identification approach, this integrated algorithm may experience a decline in prediction accuracy when the system undergoes dynamic changes, thereby leading to suboptimal control performance.

Overall, existing digital twin development algorithms typically address one or two of the fundamental aspects—modeling, synchronization, and interaction. The motivation of this study is to propose an algorithm that concurrently minimizes the initial modeling time and performs online model updating while ensuring the model fidelity and effective interactions between physical and virtual environments [13,21]. To achieve this, this paper integrates SINDYc’s framework with the extended Kalman filter (EKF)’s model parameter calibration to standardize the digital twin modeling and updating procedure while applying MPC-based control actions to promote effective interactions.

The major contributions of this paper are summarized as follows.

1. The SINDYc-based, three-step framework is utilized to identify the initial digital twin models, considering both first-principles knowledge and data-driven techniques. Implementing such a standardized, automatic identification algorithm reduces the digital twin algorithm development time while enhancing model fidelity.
2. The integration of the EKF with SINDYc’s framework enables online digital twin model updating according to real-time measurements and prevents diminishing synchronization accuracy over time.
3. The proposed EKF-based, recursive, sparse nonlinear identification is further integrated with the MPC, using the latest updated digital twin model to provide state predictions over the prediction horizon. This integration facilitates control inputs optimization while promoting effective interactions between user instructions and the system’s dynamic behavior.

## 2. Preliminaries

### 2.1. The SINDy Algorithm

When proposed, the SINDy algorithm aims to identify the nonlinear dynamics in the form of

(1) $$\begin{array}{c} \begin{array}{c} \dot{x}=f\left(x\right), \end{array} \end{array}$$

where $x=\left[\begin{array}{cccc} x_{1} & x_{2} & \cdots & x_{n_{x}} \end{array}\right]$, and it is a vector that contains all the system states. The SINDy develops a candidate feature library to involve all the features that are potential to be used to construct the dynamic model. The feature library is represented as

(2) $$\begin{array}{c} \begin{array}{c} \Theta \left(x\right)=\left[\begin{array}{cccccc} 1 & x & x⨂x & \tanh(x) & \cdots & \mathrm{FP} \end{array}\right] \end{array} \end{array}$$

where Θ(x)= is a vector that plays a symbolic role to represent the feature combinations among the system states, and FP represents the features generated from the first-principles knowledge. The x⨂x represents the product combinations among all the state variables. Then, a sparse regression problem is solved between the feature library values and the derivative of the states,

(3) $$\dot{x}=\Theta \left(x\right)\Xi ,$$

where $\Xi =\left[\begin{array}{cccc} \xi_{1} & \xi_{2} & \cdots & \xi_{n_{x}} \end{array}\right]$, representing the sparse parameter matrix. Most elements within the sparse parameter matrix are equal to zero, indicating the corresponding features are not selected to construct the system model. In this study, the SINDy algorithm is modified to solve the time-variant relationship between $x_{t}$ and $x_{t-1}$, $u_{t-1}$. The detailed modification procedure will be introduced in Section 4.

### 2.2. Extended Kalman Filter

Consider a nonlinear system with control input:

(4) $$\begin{array}{c} x_{t}=f_{t-1}\left(x_{t-1},u_{t-1}\right)+w_{t-1}, \end{array}$$

(5) $$\begin{array}{c} y_{t}=h_{t}\left(x_{t}\right)+v_{t}, \end{array}$$

where *x* denotes the system state, *u* represents the control input, *y* indicates the output measurement, and *t* corresponds to the time instant. Additionally, the process noise, *w*, and the measurement noise, *v*, are considered and assumed to be zero-mean, uncorrelated Gaussian white noise, with $Q_{\mathrm{KF}}$ and $R_{\mathrm{KF}}$ representing their respective covariance matrices. The function, *f*, captures the nonlinear relationship between the current state and the previous state and control input, while *h* denotes the nonlinear relationship between the system state and the measurement.

The EKF adheres to a two-step process comprising prediction and correction [22]. The prediction step can be described as follows:

(6) $$\begin{array}{c} {\hat{x}}_{t}^{-}=f_{t-1}\left({\hat{x}}_{t-1},u_{t-1}\right), \end{array}$$

(7) $$\begin{array}{c} P_{t}^{-}=F_{t-1}P_{t-1}F_{t-1}^{T}+Q_{\mathrm{KF}}, \end{array}$$

where ${\hat{x}}^{-}$ and $\hat{x}$ are prior and posterior state estimates, respectively. Correspondingly, $P^{-}$ and *P* represent the prior and posterior state estimation error covariance matrices. Notably, *F* denotes the Jacobian matrix of the state model, and it is computed by linearizing the nonlinear state model, *f*.

In the correction steps, first, the Kalman gain matrix, *K*, is computed as

(8) $$\begin{array}{c} K_{t}=P_{t}^{-}H_{t}^{T}{\left(H_{t}P_{t}^{-}H_{t}^{T}+R_{\mathrm{KF}}\right)}^{-1}, \end{array}$$

where *H* stands for the Jacobian matrix corresponding to the nonlinear output model, *h*. Subsequently, the innovation sequence is computed by $y_{t}-h_{t}\left({\hat{x}}_{t}^{-}\right)$. Finally, the posterior state estimate and the posterior state estimation error covariance are derived as

(9) $$\begin{array}{c} {\hat{x}}_{t}={\hat{x}}_{t}^{-}+K_{t}\left(y_{t}-h_{t}\left({\hat{x}}_{t}^{-}\right)\right), \end{array}$$

(10) $$\begin{array}{c} P_{t}=\left(I-K_{t}H_{t}\right)P_{t}^{-}. \end{array}$$

Besides state estimation, the EKF has also been employed to perform recursive parameter estimation through state vector augmentation to include the parameters as additional states. Through this augmentation, the EKF simultaneously updates both the original system states and the model parameters based on new measurements. The dual estimation capability makes the EKF an ideal tool for performing online digital twin model updating.

### 2.3. Model Predictive Control

**Figure 1:**
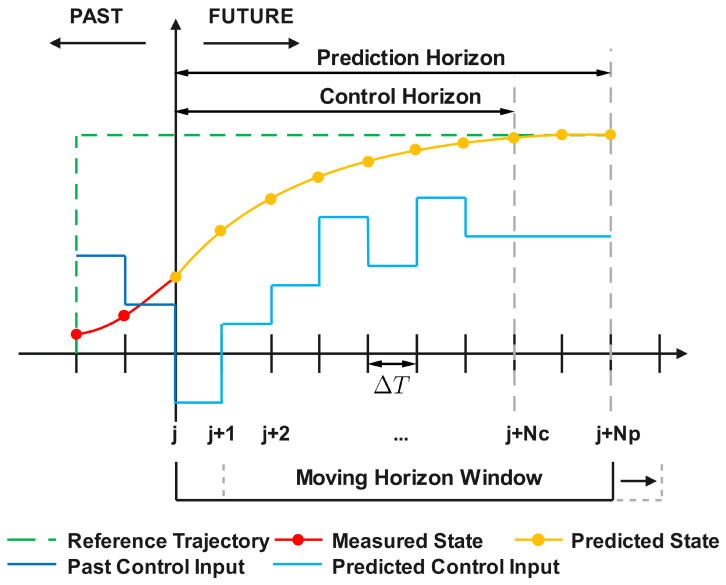
Graphical illustration of MPC.

The detailed procedure of MPC is illustrated in Figure 1. At a specific time instant *j*, several components are defined: historical states, depicted as a red dotted line, and past control inputs, represented by a black solid line. The green dashed line signifies the reference trajectory that the system state is intended to follow. Central to MPC is the determination of the control sequence, $u\left(\cdot |x_{j}\right)=\left\{u_{j+1}\ldots u_{j+N_{c}}\right\}$, based on the current state, $x_{j}$. This sequence spans the control horizon, $T_{c}=N_{c}\Delta T$, where $N_{c}$ is the number of control intervals and ΔT is the model sampling interval. This control sequence is determined by solving an optimization problem that minimizes the cost function, *J*, over the prediction horizon, $T_{p}=N_{p}\Delta T$, with $N_{p}$ representing the number of prediction intervals.

It is notable that ΔT, the model sampling interval, may differ from the measurement sampling interval. Typically, the control horizon, $T_{c}$, is equal to the prediction horizon, $T_{p}$. As a result, in this study, the condition that $N_{c}=N_{p}$ is considered. Although the control sequence over the entire control horizon is determined, only the immediate next control input, $u_{j+1}$, is implemented. Subsequently, the moving horizon window advances by one model sampling interval, leading to a reinitialization and repetition of the optimization process [23,24,25].

(11) $$\begin{array}{c} J=\sum_{t=0}^{N_{p}-1}\left({\left({\hat{x}}_{t}-r_{t}\right)}^{T}Q\left({\hat{x}}_{t}-r_{t}\right)+{\hat{u}}_{t}^{T}R_{u}{\hat{u}}_{t}\right)+\underset{terminal\;cost}{\underset{︸}{{\left({\hat{x}}_{N_{p}}-r_{N_{p}}\right)}^{T}Q_{N_{p}}\left({\hat{x}}_{N_{p}}-r_{N_{p}}\right)}} \end{array}$$

When solving the optimization problem, standing at time instant zero, the cost function *J*, shown in (11), is subject to the system dynamics and the input constraints. In (11), *r* represents the reference trajectory and $\hat{u}$ is the optimized control input, while *Q*, $Q_{N_{p}}$ and $R_{u}$ are weight matrices to perform penalization.

## 3. Problem Statement

The effectiveness of a digital twin and the performance of the MPC are intrinsically linked to the accuracy and robustness of the underlying model. First-principles models demand extensive time and process expertise to develop but are not robust under system disturbances. In contrast, data-driven models are less time-consuming to develop, but their performance heavily depends on the data quality and is unstable when the process dynamic changes. The SINDYc addresses these challenges through the development of a comprehensive feature library incorporating both first-principles knowledge and data-driven techniques [20]. The detailed feature library construction procedure is introduced in Section 4.1.

Considering (4) and (5), when applying SINDYc to identify nonlinear state dynamics, *f*, a feature library containing control input, Θ(x,u), is first constructed. Subsequently, a sparse regression problem is solved to determine the necessary features required to construct the digital twin model. The problem is formulated as follows:

(12) $$\begin{array}{c} x_{t}=\Theta \left(x_{t-1},u_{t-1}\right)\cdot \Xi_{t-1}, \end{array}$$

where $x=\left[\begin{array}{ccc} x_{1} & \ldots & x_{n_{x}} \end{array}\right]$ and $u=\left[\begin{array}{ccc} u_{1} & \ldots & u_{n_{u}} \end{array}\right]$, with $n_{x}$ and $n_{u}$ denoting the respective numbers of states and control inputs. The matrix, $\Xi \in R^{n_{\Theta }\times n_{x}}$, is defined as the sparse parameter matrix, and $n_{\Theta }$ is the number of features within the feature library.

To promote model sparsity while maintaining accuracy, an iterative sequential least squares regression algorithm is implemented in SINDYc, incorporating a thresholding parameter, λ. Parameters whose magnitudes are below this threshold are set to zero. Subsequently, another regression step is performed on the remaining active features. This iterative process leads to convergence, resulting in a sparse parameter matrix, Ξ. Additional information about solving the sparse regression problem can be found in [16,17,20].

**Figure 2:**
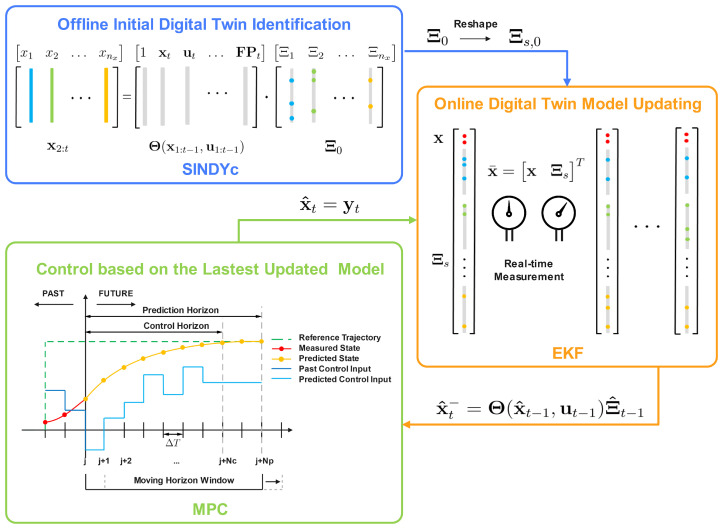
Schematic diagram of integrating the EKF-based, recursive, sparse nonlinear digital twin identification with MPC [17].

Nevertheless, the model identified by SINDYc is inherently time-invariant. Given the ever-evolving operational conditions within dynamic systems, an effective digital twin identification approach should possess the capability for online adaptability to accommodate the continuous changes in system dynamics. Therefore, a time-variant digital twin model is mandatory to continuously synchronize with the most up-to-date conditions of the system, facilitating optimal control strategy determinations. Consequently, the objective of this research is to first identify the initial digital twin model through the SINDYc algorithm and then update the model feature selections and the corresponding parameters through integration with the EKF. The SINDYc and EKF integrated recursive digital twin identification algorithm is finally integrated with MPC to enable control inputs optimization, promoting interactive capacities of digital twins. A schematic diagram of the proposed framework is shown in Figure 2.

## 4. Integration of Extended Kalman Filter-Based Recursive Sparse Nonlinear Digital Twin Identification with Model Predictive Control

In this research, the state vector of the EKF is augmented to encompass both system states and model parameters. The modified EKF is integrated with SINDYc’s framework to recursively update the digital twin model according to real-time measurements. Furthermore, to enhance model sparsity, an iterative correction procedure is incorporated within EKF’s correction step. When integrating the proposed recursive digital twin identification with MPC, the latest model is utilized to generate predictions of system states over the prediction horizon. Assuming full-state measurements, after implementing the first element of the MPC-determined optimal control sequence, the received measurement is utilized as the initial estimate of the system state in the next model sampling instant.

### 4.1. Feature Library Construction and Initial Identification

In the initialization phase of the proposed algorithm, historical data, $x_{1:t}$ and $u_{1:t}$, are collected, where the control input, u, is assumed to be able to excite the system. Subsequently, a feature library can be established in the following format,

(13) $$\begin{array}{c} \Theta \left(x,u\right)=\left[\begin{array}{ccccccc} 1 & x & u & x⨂x & x⨂u & \ldots & \mathrm{FP} \end{array}\right], \end{array}$$

where the symbol ⨂ indicates the product combination among all the variables within the two specified vectors. In addition to the polynomial combinations, more data-driven features could be included, such as trigonometric terms, sin(x), tanh(x), cos(x⨂u), and the sigmoid function, $\frac{1}{1+e^{-x}}$. Meanwhile, FP signifies the terms generated based on the first-principles information. For example, when modeling the heat transfer of the humid air flow passing through a desiccant wheel, the heat adsorption of the desiccant material is described by

(14) $$\begin{array}{c} \begin{array}{c} Q=h_{v}\left(1+0.2843e^{-10.28W}\right) \end{array}, \end{array}$$

where Q represents the heat adsorbed by the regular density silica gel (desiccant material of the wheel), $h_{v}$ is the evaporation latent heat of water, and *W* is a process variable indicating the water content of the desiccant material. As a result, when identifying the digital twin model of a rotary desiccant dehumidifier, the feature, $e^{-10.28W}$, can be included in the feature library of the SINDy-based algorithm.

The construction of a feature library usually starts with the inclusion of simple polynomial features and first-principles features. Subsequently, the library complexity is gradually increased if the existing features are insufficient to achieve satisfactory performance. To prevent overfitting, when determining the initial nonlinear state model, *f*, a sparse regression problem between $x_{2:t}$ and $\Theta (x_{1:t-1},u_{1:t-1})$, is solved, generating the initial sparse parameter matrix, $\Xi_{0}$. Later, during the EKF-based online digital twin model updating, a sparsity-promoting correction step is incorporated, which will be further discussed in Section 4.3.

### 4.2. Extended Kalman Filter Vectors Augmentation

To facilitate the recursive, simultaneous estimation of system states and digital twin model parameters, the proposed algorithm augments the state vector within the EKF algorithm as, $\bar{x}={\left[\begin{array}{cc} x & \Xi_{s} \end{array}\right]}^{T}$. When constructing the one-dimensional $\bar{x}$, the two-dimensional Ξ, is reshaped into $\Xi_{s}$. Specifically,

(15) $$\begin{array}{c} \Xi_{s}={\left[\begin{array}{cccc} \Xi_{1} & \Xi_{2} & \ldots & \Xi_{n_{x}} \end{array}\right]}^{T}. \end{array}$$

In (15), for i=1 to $n_{x}$, $\Xi_{i}$ denotes the parameter vector corresponding to each individual state variable. In specific,

(16) $$\begin{array}{c} \Xi_{i}=\left[\begin{array}{cccc} \xi_{i,1} & \xi_{i,2} & \ldots & \xi_{i,n_{\Theta }} \end{array}\right], \end{array}$$

where ξ represents the model parameter corresponding to each feature for a specific system state. Furthermore, $n_{\Xi }=n_{x}\times n_{\Theta }$, representing the total number of elements within the sparse parameter matrix.

In this context, the state prediction step of the EKF is modified as follows:

(17) $$\begin{array}{cc} {\hat{\bar{x}}}_{t}^{-} & =\left[\begin{array}{c} f_{t-1}\left({\hat{x}}_{t-1},u_{t-1}\right)\\ I\cdot {\hat{\Xi }}_{s,t-1} \end{array}\right]=\left[\begin{array}{c} \Theta \left({\hat{x}}_{t-1},u_{t-1}\right){\hat{\Xi }}_{t-1}\\ {\hat{\Xi }}_{s,t-1} \end{array}\right]\\ & ={\left[\begin{array}{cc} {\hat{x}}_{t}^{-} & {\hat{\Xi }}_{s,t}^{-} \end{array}\right]}^{T}, \end{array}$$

where ${\hat{\bar{x}}}_{t}^{-}\in R^{n_{s}}$ represents the augmented prior state estimate and $n_{s}=n_{x}+n_{\Xi }$. In this step, the random walk model is employed to predict the digital twin model parameters at the next model sampling instant. Consequently, the prior model parameter estimate, ${\hat{\Xi }}_{s,t}^{-}$, remains equal to the posterior model parameter estimate from the last model sampling instant.

Accordingly, the prediction of the state estimation error covariance matrix is augmented as

(18) $$\begin{array}{c} {\bar{P}}_{t}^{-}={\bar{F}}_{t-1}{\bar{P}}_{t-1}{\bar{F}}_{t-1}^{T}+{\bar{Q}}_{\mathrm{KF}}, \end{array}$$

where ${\bar{P}}_{t}^{-}\in R^{n_{s}\times n_{s}}$, and ${\bar{Q}}_{\mathrm{KF}}\in R^{n_{s}\times n_{s}}$ denoting the augmented process noise covariance matrix. Additionally, $\bar{F}$ signifies the augmented Jacobian matrix of the nonlinear state model,

(19) $$\begin{array}{cc} {\bar{F}}_{t-1} & ={\left[\begin{array}{cc} \frac{\partial f}{\partial x}{|}_{{\hat{\bar{x}}}_{t-1},u_{t-1}} & \frac{\partial f}{\partial \Xi_{s}}{|}_{{\hat{\bar{x}}}_{t-1},u_{t-1}}\\ 0 & 0 \end{array}\right]}_{n_{s}\times n_{s}}, \end{array}$$

where

(20) $$\begin{array}{cc} \frac{\partial f}{\partial x}{|}_{{\hat{\bar{x}}}_{t-1},u_{t-1}} & =\frac{\partial (\Theta \left({\hat{x}}_{t-1},u_{t-1}\right){\hat{\Xi }}_{t-1})}{\partial x}{|}_{{\hat{\bar{x}}}_{t-1},u_{t-1}}\\ & =\frac{\partial (\Theta \left({\hat{x}}_{t-1},u_{t-1}\right))}{\partial x}{\hat{\Xi }}_{t-1}, \end{array}$$

and

(21) $$\begin{array}{cc} \frac{\partial f}{\partial \Xi_{s}}{|}_{{\hat{\bar{x}}}_{t-1},u_{t-1}} & =\frac{\partial (\Theta \left({\hat{x}}_{t-1},u_{t-1}\right){\hat{\Xi }}_{t-1})}{\partial \Xi_{s}}{|}_{{\hat{\bar{x}}}_{t-1},u_{t-1}}\\ & =\Theta ({\hat{x}}_{t-1},u_{t-1}). \end{array}$$

### 4.3. Sparsity-Promoting Correction

In MPC applications, typically, the availability of full-state measurement can be assumed. Under this assumption, $y_{t}=x_{t}$ and ${\hat{y}}_{t}={\hat{x}}_{t}^{-}$ are employed in the subsequent derivations, and the state observability and controllability are assumed in this study. During the correction process, the augmented Kalman gain matrix, ${\bar{K}}_{t}$, is calculated as

(22) $$\begin{array}{cc} {\bar{K}}_{t} & ={\bar{P}}_{t}^{-}{\bar{H}}_{t}^{T}{\left({\bar{H}}_{t}{\bar{P}}_{t}^{-}{\bar{H}}_{t}^{T}+R_{\mathrm{KF}}\right)}^{-1}, \end{array}$$

and the augmented Jacobian matrix, ${\bar{H}}_{t}$, is computed as

(23) $$\begin{array}{c} {\bar{H}}_{t}={\left[\begin{array}{cc} \frac{\partial h}{\partial x}{|}_{{\hat{\bar{x}}}_{t}^{-}} & \frac{\partial h}{\partial \Xi_{s}}{|}_{{\hat{\bar{x}}}_{t}^{-}} \end{array}\right]}_{n_{x}\times n_{s}}, \end{array}$$

where

(24) $$\begin{array}{cc} \frac{\partial h}{\partial x}{|}_{{\hat{\bar{x}}}_{t}^{-}} & =\frac{\partial {\hat{x}}_{t}^{-}}{\partial {\hat{x}}_{t}^{-}}=1, \end{array}$$

and

(25) $$\begin{array}{cc} \frac{\partial h}{\partial \Xi_{s}}{|}_{{\hat{\bar{x}}}_{t}^{-}} & =\frac{\partial {\hat{x}}_{t}^{-}}{\partial {\hat{\Xi }}_{s,t}^{-}}{|}_{{\hat{\bar{x}}}_{t}^{-}}\\ & =\frac{\partial (\Theta \left({\hat{x}}_{t-1},u_{t-1}\right){\hat{\Xi }}_{t-1})}{\partial {\hat{\Xi }}_{s,t-1}}\\ & =\Theta ({\hat{x}}_{t-1},u_{t-1}). \end{array}$$

Subsequently, the correction step promotes model sparsity through an iterative procedure. The value of the thresholding parameter, λ, is incrementally increased until a noticeable decline in the model performance is observed. Within this context, elements of the prior estimate of the single-dimensional sparse parameter matrix, ${\hat{\Xi }}_{s,t}^{-}$, that exhibit magnitudes below this threshold are set to zero. Following this, the sparsity-enhanced ${\hat{\Xi }}_{s,t}^{-}$ is reshaped into the two-dimensional sparse parameter matrix, ${\hat{\Xi }}_{t}^{-}$, which is then utilized to generate new prior state variable estimates, denoted as ${\hat{x}}_{t}^{\prime -}$. The new state variable estimates, together with the sparsity-enhanced ${\hat{\Xi }}_{s,t}^{-}$ will form the new augmented state vector, ${\hat{\bar{x}}}_{t}^{\prime -}$.

The innovation sequence contributes to the correction of the new augmented state vector, ${\hat{\bar{x}}}_{t}^{\prime -}$, generating the posterior augmented state vector, ${\hat{\bar{x}}}_{t}$. The posterior estimate of the augmented state vector then replaces the ${\hat{\bar{x}}}_{t}^{\prime -}$ and is subject to another sparsity-promoting correction iteration. Typically, convergence is achievable through five to ten iterations. Therefore, the iteration parameter, *k*, can be set to ten for effective convergence. The overall sparsity-promoting correction procedure can be formulated as follows:

(26) $$\begin{array}{cc} \mathrm{for}\; & q=1:k,\\ & \left|{\hat{\Xi }}_{s,t}^{-}\right|<\lambda =0,\\ & {\hat{x}}_{t}^{\prime -}=\Theta \left({\hat{x}}_{t-1},u_{t-1}\right){\hat{\Xi }}_{t}^{-},\\ & {\hat{y}}_{t}={\hat{x}}_{t}^{\prime -},\\ & {\hat{\bar{x}}}_{t}={\hat{\bar{x}}}_{t}^{\prime -}+{\bar{K}}_{t}\left(y_{t}-{\hat{y}}_{t}\right),\\ & {\hat{\bar{x}}}_{t}^{\prime -}={\hat{\bar{x}}}_{t}.\\ \mathrm{end} \end{array}$$

Eventually, the sparse parameter matrix, ${\hat{\Xi }}_{t}^{-}$, determined from the final iteration, is employed to generate the final posterior estimates of the augmented state vector so as to ensure the model sparsity and the accuracy of posterior state estimates. Furthermore, the augmented posterior state estimation error covariance matrix is

(27) $$\begin{array}{cc} {\bar{P}}_{t} & =\left(I-{\bar{K}}_{t}{\bar{H}}_{t}\right){\bar{P}}_{t}^{-}. \end{array}$$

### 4.4. Integration with Model Predictive Control

The initial identification of $\Xi_{0}$ through the SINDYc provides the foundational estimates for the digital twin model parameters. Afterward, the online adjustments of the model features and the corresponding parameters are performed upon receiving the newly acquired measurement at each model sampling instant. To prevent the trivial solution, $x_{t}=x_{t-1}$, the model sampling interval is usually greater than the measurement sampling interval. The real-time data will form the feature library values at each model sampling instant to contribute to the digital twin online modification.

**Figure 3:**
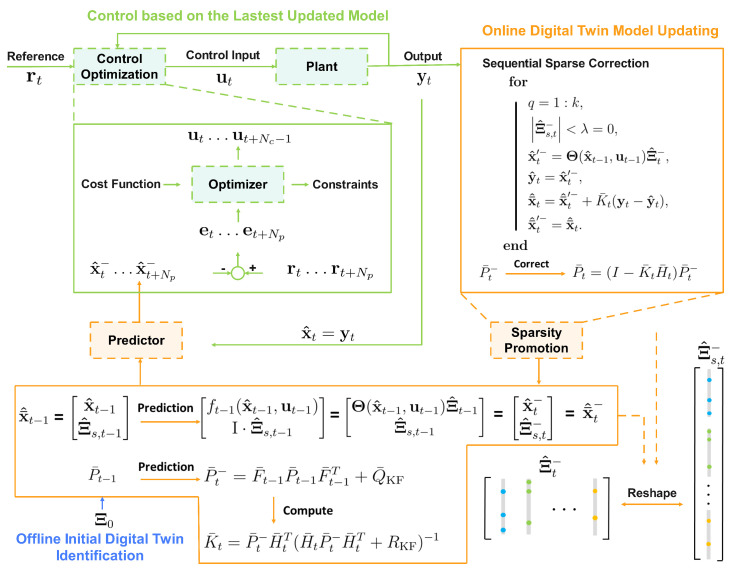
Graphical illustration of integrating the EKF-based, recursive, sparse nonlinear digital twin identification with MPC.

During the MPC execution, weight matrices, $Q,\;Q_{N_{p}},$ and $R_{u}$, are predetermined according to control specifications. In the proposed, integrated, recursive digital twin identification with MPC approach, the latest updated model is utilized to forecast system states over the prediction horizon, where the model remains static. The predicted states are subsequently utilized to compute the optimal control sequence according to (11). Once the initial component of the optimal control sequence has been implemented, full-state measurements, $y_{t}$, become available. These measurements are utilized in the sparsity-promoting correction procedure to update the system state estimates and sparse model parameters. Following this correction phase, the full-state measurements are then utilized as the initial state estimates, ${\hat{x}}_{t}$, for the next model sampling instant. The main steps of the proposed algorithm are shown in Algorithm 1 and the corresponding graphical representation is provided in Figure 3.

It is notable that the SINDy-based identifications are readily applicable to large-scale applications through expanding the comprehensive feature libraries. By emphasizing sparsity promotion, SINDy-based frameworks facilitate the identification of parsimonious digital twin models for large-scale industrial processes. In the context of MPC implementations, the large MIMO control problems are typically decomposed into multiple multi-input, single-output (MISO) problems or simpler MIMO configurations. This decomposition is essential because the complexity of variable interactions in large-scale MPC strategies. Consequently, when solving large-scale control problems, the proposed recursive digital twin identification with the control algorithm can be deployed in parallel to efficiently achieve the desired control objectives.
**Algorithm 1: ** Integration of EKF-based, Recursive, Sparse Nonlinear Digital Twin Identification with MPC     **Data:**Feature library, Θ(x,u);
Process noise and measurement noise covariances, ${\bar{Q}}_{\mathrm{KF}},R_{\mathrm{KF}}$;
MPC weight matrices, $Q,Q_{N_{p}},R_{u}$;
Initial sparse parameter matrix from SINDYc, $\Xi_{0}$;
Initialize process state, $x_{0}$, state estimation error covariance, ${\bar{P}}_{0}$, and control 
input, $u_{0}$.     **Result:** Optimal control sequence, $u^{*}$.     **for*** each model sampling instant, t*** do**((
 Cost function, J=0
  **if**
*$i⩽N_{p}$*
**then**


Predict the states within the prediction horizon:


(28)$$\begin{array}{c} {\hat{x}}_{i}^{-}=f_{t-1}\left({\hat{x}}_{i-1},u_{i-1}\right) \end{array}.$$


Compute cumulative cost function, *J*;


Solve the optimization problem, and compute the optimal control


sequence, $u^{*}$;
  
**end**

  Apply the first element of the optimal control sequence.
  Obtain full-state measurements, $y_{t}$.
  Update model parameter estimates, ${\hat{\Xi }}_{t}^{-}$.
  Utilize $y_{t}$ as the initial state estimate, ${\hat{x}}_{t}$, in the next model sampling instant.
     **end**


## 5. Case Study

In this section, the prediction accuracy of the proposed EKF-based recursive sparse nonlinear digital twin identification algorithm is evaluated through an industrial diesel characteristic prediction case study, and the performance is compared with other advanced approaches. Two numerical simulation examples are used to demonstrate the control performance of the proposed recursive digital twin identification with control algorithm against system dynamic changes and training data noise.

### 5.1. Diesel Characteristics Prediction Based on the Digital Twin Model

The diesel hydrotreating (DHT) unit plays a critical role in enhancing fuel performance and meeting regulatory requirements for transportation and industrial applications [26]. Within the DHT unit, the feed streams are initially preheated in the furnace before being fed into the reactor, where key processes such as hydrodesulfurization, hydrodenitrogenation, and hydrocracking occur in the presence of hydrogen and catalysts. The output from the reactor then enters the separator for preliminary separation of lighter and heavier products. The lighter reaction products then proceed to an absorber for sulfur and ammonia removal. The heavier products are further processed in a fractionation tower, where additional separations take place to yield light hydrocarbons, gasoline, jet fuel, diesel, and heavy bottom products [27,28].

**Figure 4:**
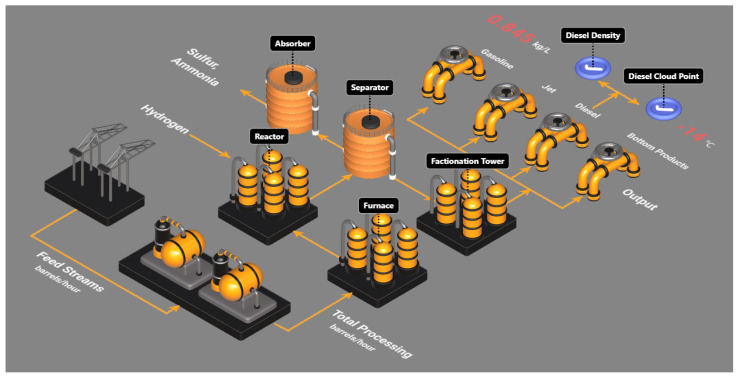
Graphical illustration of the DHT digital twin.

The distillation process within the DHT unit ensures that the final diesel product meets the essential quality specifications, such as sulfur content and cetane number. Among these quality parameters, diesel density and diesel cloud point require laboratory measurements, which are often conducted infrequently. To address this limitation, the proposed EKF-based recursive digital twin identification algorithm is used to construct and update the digital twin model, to provide frequent predictions of these two quality values. When evaluating the prediction accuracy, the reference lab measurements are assumed to be available at three different frequencies. Figure 4 shows the digital twin for the DHT unit. The z-score normalization is applied to the industrial data during the comparative analysis for proprietary reasons.

Production hourly data over a ten-month period is used to construct the digital twin model and test the prediction accuracy. To prevent the trivial solution, the model sampling interval is selected as every 24 h, which gives 295 total samples. To benchmark the performance of the proposed algorithm, two contrasting model identification frameworks are evaluated. First, the dynamic mode decomposition with control (DMDc) is implemented to assess whether a simpler, linear identification method can achieve comparable or superior results. Conversely, the neural network with control (NNc) is tested to determine if a more complex, nonlinear model yields significant performance gains. This comparative analysis evaluates the proposed algorithm within the spectrum of model complexity and fidelity. The first 110 samples are used as the training data to identify the time-invariant digital twin model, and the remaining 185 modeling samples are used to test the prediction accuracy of the analyzed approaches.

To realize effective control, one of the critical steps is to select the control input, *u*, which is also called manipulated variable (MV). It serves as the primary actuation mechanism applied to a physical plant. By strategically adjusting the control input, based on the designed control logic, the controller drives the system toward its desired performance. According to the first-principles knowledge, the reactor inlet temperature plays a significant role in affecting the diesel density and cloud point [29,30]. Consequently, the reactor inlet temperature in the DHT unit is selected as the control input. A second-order polynomial and trigonometric integrated feature library is constructed for both SINDYc and the proposed algorithm,

(29) $$\begin{array}{c} \Theta \left(x,u\right)=\left[\begin{array}{cccccc} 1 & x & u & x⨂x & \sin(x) & \tanh(x) \end{array}\right]. \end{array}$$

In the meanwhile, the thresholding parameter, λ, is set to 0.1.

During the test period, the full-state measurements are assumed to be available at three different model sampling intervals to assess the proposed algorithm’s prediction accuracy within the prediction horizon. In addition, when implementing the proposed algorithm, ${\bar{Q}}_{\mathrm{KF}}=\mathrm{diag}\left\{0.2\;0.4\;1e^{-4}\ldots 1e^{-4}\right\}$, $R_{\mathrm{KF}}=\mathrm{diag}\left\{0.4\;0.3\right\}$, and ${\bar{P}}_{0}=\mathrm{diag}\left\{1e^{-4}\;\ldots \;1e^{-4}\right\}$. Furthermore, a nonlinear autoregressive neural network with external input is used for the NNc, with one hidden layer containing eight neurons to achieve an optimal balance between NNc’s model accuracy and complexity.

The prediction mean squared errors (MSEs) of the algorithms considered, based on the 185 test samples, are presented in Table 1. In the right portion of Table 1, the MSE results for the proposed algorithm are provided, with reference lab measurements available at intervals of three, five, and seven days. According to the results presented in Table 1, the DMDc algorithm, which uses linear representations to approximate nonlinear systems, experiences reduced accuracy due to the complex nonlinearities and dynamic behavior changes inherent in the diesel characteristics. In addition, while the NNc algorithm is capable of handling the process nonlinearities, its performance is limited by the relatively small sample size. Of the three time-invariant algorithms considered, SINDYc outperforms the others, which shows its ability to effectively manage nonlinearities while requiring relatively fewer samples. 

Across all three reference measurement availabilities, the proposed algorithm consistently outperforms SINDYc in terms of prediction accuracy. Specifically, the proposed algorithm reduces the MSE from 0.43 to 0.36 and 0.64 to 0.48 by updating the model only once per week, which improves prediction accuracy by 16.3% and 24.9% over SINDYc. This significant improvement in prediction accuracy highlights the advantage of integrating the SINDYc with the EKF to maintain the digital twin synchronization precision over time.

### 5.2. Simulation Examples

#### 5.2.1. The Lotka–Volterra System

The Lotka–Volterra system is a two-dimensional ordinary differential equation (ODE) system describing the interactive dynamics between coexisting predator and prey populations within an ecosystem. The dynamics of the prey population, $x_{1}$, and the predator population, $x_{2}$, are mathematically expressed as follows [31]:

(30) $$\begin{array}{c} {\dot{x}}_{1}=\alpha x_{1}-\beta x_{1}x_{2} \end{array},$$

(31) $$\begin{array}{c} {\dot{x}}_{2}=-\gamma x_{2}+\delta x_{1}x_{2}+u \end{array},$$

where α corresponds to the prey population growth rate in the absence of predators; β characterizes the impact of the predator’s presence on the prey’s growth rate; γ represents the mortality rate of predators in the absence of their prey; and δ signifies the growth rate of the predator when consuming the prey. When implementing the proposed algorithm to perform identification and control for the given ODE system, the discrete–discrete variant method, as outlined in [32], is employed. This approach discretizes the continuous models using the fourth-order Runge–Kutta (RK4) algorithm, facilitating the application of the proposed algorithm.

The system parameter values are altered twice throughout the simulation period. The simulation duration, *T*, is set to 100. Since this is a simulated ODE system, the model sampling interval and measurement sampling interval are the same, denoted as Δt=0.1, resulting in a total of 1000 sampling intervals. For the initial 300 sampling intervals, α,β,γ,δ are established as 0.5,0.025,0.5,0.02 and are adjusted to 0.4,0.02,0.4,0.02 in the next 300 sampling intervals. In the final 400 sampling intervals, the parameters are further modified to 0.3,0.015,0.3,0.03. In addition, the values of control inputs are constrained to $\left[\begin{array}{c} -10,10 \end{array}\right]$. The initial states of prey and predator populations are set at 40 and 30, with λ=0.01 and $N_{p}=N_{c}=6$. The MPC weight matrices are configured as $Q=Q_{N_{p}}=\mathrm{diag}\left\{0.7\;0.7\right\}$ and $R_{u}=0.7$. The control objective is to stabilize populations of prey and predator to their respective targeting values, $\frac{\gamma }{\delta }$ and $\frac{\alpha }{\beta }$.

During the evaluation procedure, both MPC-based algorithms and reinforcement learning (RL)-based methods are analyzed for performance comparison. In the MPC implementations, models identified through the DMDc, NNc, SINDYc, and the proposed EKF-based, recursive, sparse nonlinear identification are utilized. In the RL implementations, the multi-layer perceptron (MLP) policy is employed with two optimization techniques: the on-policy, proximal policy optimization (PPO) and the off-policy, soft actor–critic (SAC).

When implementing the MPC-based algorithms, noise with a magnitude equivalent to 10% of the standard deviation of the clean data is incorporated into the training dataset. Additionally, the initial 100 samples are utilized to identify the time-invariant system models for the DMDc, SINDYc, and the proposed algorithm. In contrast, the NNc requires 400 training samples to achieve stable control performance. The control input used to excite the system for training is

(32) $$\begin{array}{c} u={\left(2\sin\left(t\right)+\sin\left(0.1t\right)\right)}^{2}. \end{array}$$

An identical polynomial feature library is constructed for both SINDYc and the proposed algorithm,

(33) $$\begin{array}{c} \Theta \left(x,u\right)=\left[\begin{array}{cccc} 1 & x & u & x⨂x \end{array}\right]. \end{array}$$

In addition, it takes 21.7, 39.1, 219.7, and 1608.6 s for DMDc, SINDYc, the proposed algorithm, and NNc to accomplish the MPC implementations, respectively. The DMDc, being a simplified version of the SINDYc, demands less computational time. In contrast, the NNc requires a larger training sample size and longer computational time. Since the proposed algorithm updates the digital twin model at every model sampling instant, its overall computational time is longer than that of DMDc and SINDYc. However, this time-variant approach is significantly more computationally efficient for real-time implementations compared to the need for recomputing the model using time-invariant approaches at each model sampling step.

**Figure 5:**
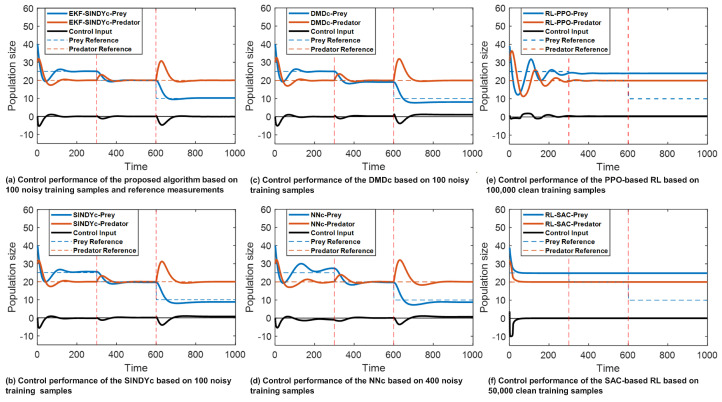
The Lotka–Volterra system control performance comparison.

In the context of RL implementations, the PPO and SAC algorithms require 100,000 and 50,000 clean samples, respectively, to achieve stable and accurate control performance for the initial control objectives, necessitating the generation of additional samples. The corresponding training durations are 206.4 s for PPO and 1168.8 s for SAC. Although the SAC method requires fewer training samples, it is an off-policy method that utilizes replay buffer sampling to optimize the control policy, which results in longer training time. The control performance of these analyzed algorithms is shown graphically in Figure 5, where the proposed algorithm is referred to as the EKF-SINDYc.

As observed in Figure 5a–d, owing to the system dynamic changes and the noise added to the training dataset, NNc, DMDc, and SINDYc all show deviations in controlling the prey population to its objectives. By incorporating the reference measurements over time, the proposed algorithm successfully controls the prey and predator’s populations to their respective targets even under system dynamic changes and initial model inaccuracy.

For the RL-based algorithms, clean data are used. According to Figure 5e,f, the PPO-optimized policy requires a relatively longer settling time and only achieves stable control results after the first simulation period. In contrast, the SAC-optimized policy is capable of reaching quick and stable control results without exhibiting overshooting or oscillations. However, the performance of both RL-based algorithms strongly depends on the sample volume and quality, as well as the training environment, which limits their ability to adapt to new control objectives after the initial 300 sampling intervals. Consequently, due to their extended training duration and limited adaptability to dynamic changes, RL-based algorithms are less suited for real-time digital twin applications compared to MPC-based algorithms under current computational constraints.

Overall, the proposed algorithm maintains effective control over both prey and predator populations across the three simulation periods. This shows the proposed algorithm’s robustness to initial model defects and capability to adaptively modify the digital twin models in response to changes in system dynamics, which achieves more effective control performance and ensures stronger interactive capacities.

#### 5.2.2. The Discrete-Time System

In the second simulation example, a discrete-time system is given as follows:

(34) $$x_{1,t}=a\tanh\left(x_{1,t-1}\right)-b\sin\left(x_{2,t-1}\right)+c\cos\left(x_{1,t-1}\right)+du_{t-1},$$

(35) $$\begin{array}{c} x_{2,t}=e\cos\left(x_{1,t-1}\right)+f\sin\left(x_{2,t-1}\right)+gu_{t-1} \end{array},$$

where a,b,c,d,e,f,g are model parameters. The simulation duration, *T*, is defined as 100, with a measurement sampling interval of 0.1. The model sampling interval takes every five measurement sampling intervals, which yields Δt=0.5, and a total of 200 model sampling intervals. For the initial 100 model sampling intervals, the parameter values are set as 3.8,3,1,1.1,0.5,0.85,1 and are adjusted to 4,2.9,1,1,0.5,1,1.2 in the final 100 model sampling intervals. The control objective is to guide the system states towards their respective target values, $\frac{a}{fd}$ and bg.

Owing to the limited sample numbers, in this example, the performance of the DMDc and SINDYc is compared with the proposed algorithm. For the initial system identification, 80 samples are provided at two distinct noise levels, corresponding to 7.5%, and 10% relative to the standard deviation of the clean data. The feature library for SINDYc and the proposed algorithm is

(36) $$\begin{array}{c} \Theta \left(x,u\right)=\left[\begin{array}{ccccccc} 1 & x & u & x⨂x & \sin(x) & \cos(x) & \tanh(x) \end{array}\right]. \end{array}$$

To rigorously evaluate the algorithms’ performance, 10,000 Monte Carlo simulation runs are conducted with $x_{0}=\left[\begin{array}{cc} 3 & 2 \end{array}\right]$, λ=0.4, and $N_{c}=N_{p}=5$. The MPC weight matrices are set as $Q=Q_{N_{p}}=\mathrm{diag}\left\{0.9\;0.9\right\}$, $R_{u}=0.95$.

**Figure 6:**
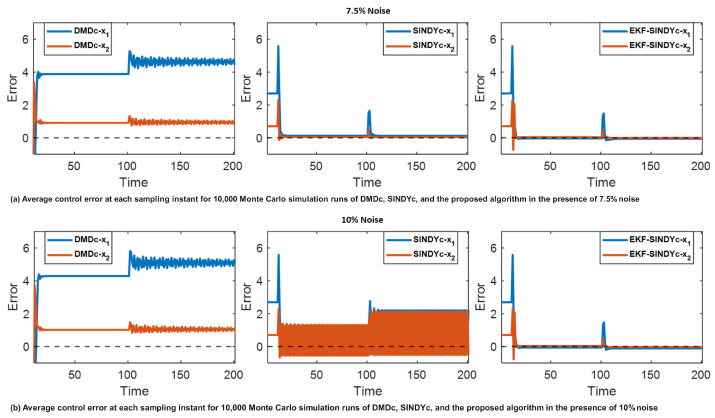
Monte Carlo average error plots based on the control performance of DMDc, SINDYc, and the proposed algorithm.

Figure 6 shows the average error between the reference trajectories and the state trajectories controlled by the evaluated algorithms. This comparison is based on the 10,000 Monte Carlo simulation runs conducted under two distinct noise levels. The Monte Carlo test takes the average error of the 10,000 simulation runs at each time step. To provide a more straightforward comparison, the average Monte Carlo errors of the overall 200 simulation time steps are given in Table 2.

It can be observed from Figure 6a,b and Table 2 that the DMDc shows larger errors in controlling the states to their corresponding objectives and becomes unstable after the system dynamic changes. The inferior performance of DMDc is attributable to its limited capacity for managing nonlinearities and system dynamic changes. Additionally, while SINDYc can achieve relatively lower control errors, its performance becomes unstable when the noise level increases to 10%, and it exhibits larger control errors after changes in system dynamics.

In contrast, the proposed EKF-based recursive sparse nonlinear digital twin identification with MPC algorithm consistently demonstrates stable control performance and reduced average error among the three algorithms considered. This result further shows that the proposed algorithm is robust to both system dynamic changes and the initial model defects arising from the training data noise. By including real-time measurements to recursively adjust the digital twin model online, the proposed algorithm achieves enhanced control performance.

## 6. Conclusions

The digitalization of industrial production processes has emerged as an inevitable trend in recent years. In this procedure, the digital twin plays a significant role, serving as the bridge between the physical system and the digital world. This study focuses on the modeling, synchronization, and interaction perspectives of the digital twin’s underlying algorithm. The proposed algorithm first utilizes SINDYc to streamline the initial digital twin model identification procedure, saving the model development time while maintaining model precision. Subsequently, the EKF algorithm is integrated with the SINDYc to recursively update the selection of model features and their corresponding parameters to ensure precise synchronization between the digital twin model and the physical operations. Finally, by integrating the proposed adaptive digital twin identification with the MPC, enhanced control performance is achieved, promoting the effective interaction between user instructions and system dynamics. The comparative results from the industrial case study and simulation examples demonstrate that the proposed algorithm is robust to both training data noise and system dynamic changes, making it well-suited for performing online digital twin identification with control. The proposed recursive digital twin modeling with control algorithm is applicable across a wide range of applications by specifying corresponding first-principles features of different domains, such as automobile manufacturing, disease infection modeling, and urban planning. Future research can be conducted to facilitate the development and implementation of the complete digital twin life cycle in diverse areas and perform necessary modifications.

## Figures and Tables

**Table 1:** Prediction error comparison in terms of MSE.

Prediction Objective	Approaches	MSE	EKF-SINDYc MSE		
			Prediction Horizon		
			3	5	7
Diesel Density	NNc	0.85			
	DMDc	0.53	0.12	0.28	0.36
	SINDYc	0.43			
Cloud Point	NNc	0.87			
	DMDc	1.48	0.29	0.42	0.48
	SINDYc	0.64			

**Table 2:** Monte Carlo error overall average.

Noise Level	Approaches	$x_{1}$ Average Error	$x_{2}$ Average Error
7.5% Noise	DMDc	3.712	0.499
	SINDYc	0.319	0.101
	EKF-SINDYc	0.127	0.059
10% Noise	DMDc	4.108	0.562
	SINDYc	1.125	0.612
	EKF-SINDYc	0.157	0.068

## Data Availability

The datasets presented in this article are not readily available because this research used the industrial data, which is confidential.

## Abbreviations

The following abbreviations are used in this manuscript:

APC	Advanced process control
EKF	Extended Kalman filter
DHT	Diesel hydrotreating
DMDc	Dynamic mode decomposition with control
ICT	Information and communications technology
RL	Reinforcement learning
RTO	Real-time optimization
MIMO	Multi-input, multi output
MISO	Multi-input, single output
MPC	Model predictive control
MSE	Mean squared error
MV	manipulated variable
NNc	Neural network with control
ODE	Ordinary differential equation
PLC	Programmable logic controller
PPO	Proximal policy optimization
SAC	Soft actor-critic
SINDy	Sparse identification of nonlinear dynamics
SINDYc	Sparse identification of nonlinear dynamics with control
